# One-step sputtering of MoSSe metastable phase as thin film and predicted thermodynamic stability by computational methods

**DOI:** 10.1038/s41598-024-57243-3

**Published:** 2024-03-26

**Authors:** Oscar A. López-Galán, Torben Boll, John Nogan, Delphine Chassaing, Alexander Welle, Martin Heilmaier, Manuel Ramos

**Affiliations:** 1 Institute of Nanotechnology (INT), Karlsruhe Institute of Technology (KIT), Hermann-von-Helmholtz-Platz 1, 76344 Eggenstein-Leopoldshafen, Germany; 2 Institute for Applied Materials and Materials Science (IAM-WK), Karlsruhe Institute of Technology (KIT), Engelbert-Arnold-Str. 4, 76131 Karlsruhe, Germany; 3 Karlsruhe Nano Micro Facility (KNMF), Karlsruhe Institute of Technology (KIT), Hermann-von-Helmholtz-Platz 1, 76344 Eggenstein-Leopoldshafen, Germany; 4 Sandia National Laboratories, Center for Integrated Nanotechnologies (CINT), 1101 Eubank Bldg. SE, Albuquerque, NM 87110 USA; 5 Institute of Functional Interfaces (IFG), Karlsruhe Institute of Technology (KIT), Hermann-von Helmholtz-Platz 1, 76344 Eggenstein-Leopoldshafen, Germany; 6 Departamento de Física y Matemáticas, Instituto de Ingeniería y Tecnología, Universidad Autónoma de Ciudad Juárez, Avenida del Charro #450 N, Ciudad Juárez, 32310 CHIH México

**Keywords:** Electronic devices, Electronic structure

## Abstract

We present the fabrication of a MoS_2−x_Se_x_ thin film from a co-sputtering process using MoS_2_ and MoSe_2_ commercial targets with 99.9% purity. The sputtering of the MoS_2_ and MoSe_2_ was carried out using a straight and low-cost magnetron radio frequency sputtering recipe to achieve a MoS_2−x_Se_x_ phase with x = 1 and sharp interface formation as confirmed by Raman spectroscopy, time-of-flight secondary ion mass spectroscopy, and cross-sectional scanning electron microscopy. The sulfur and selenium atoms prefer to distribute randomly at the octahedral geometry of molybdenum inside the MoS_2−x_Se_x_ thin film, indicated by a blue shift in the A_1g_ and E^1^_g_ vibrational modes at 355 cm^−1^ and 255 cm^−1^, respectively. This work is complemented by computing the thermodynamic stability of a MoS_2−x_Se_x_ phase whereby density functional theory up to a maximum selenium concentration of 33.33 at.% in both a Janus-like and random distribution. Although the Janus-like and the random structures are in the same metastable state, the Janus-like structure is hindered by an energy barrier below selenium concentrations of 8 at.%. This research highlights the potential of transition metal dichalcogenides in mixed phases and the need for further exploration employing low-energy, large-scale methods to improve the materials’ fabrication and target latent applications of such structures.

## Introduction

Layered molybdenum disulfide (MoS_2_) has been used as a semiconductor in the improvement of thin films for the development of hybrid solar cell prototypes. When mixing the material with organic molecules, the array 2H-MoS_2_/*p*-type organic semiconductor can achieve conversion efficiencies of up to 2.8%^1^. A recent study highlights remarkable mechanical properties of MoS_2_, denoted by a reported elastic modulus of E = 136 ± 2 GPa and hardness of H = 10.5 ± 0.1 GPa^2^. The fabrication by radio-frequency sputtering (RF-sputtering) reveals a vertical crystal growth alignment along the [101] direction corresponding to laminar colonies of grains. Moreover, recent advances in tuning the electronic and optical behavior of transition metal dichalcogenides (TMDC) by doping^3,4^ and heterostructure formation^5,6^ have expanded the interest in TMDC even further. From the approaches mentioned, heterostructure formation has stood as a potential route to attain new mixed phases with enhanced piezoelectric properties^7^, improved catalytic activity^8^, band gap engineering^9^, and reduced contact resistance. The latter, for example, has been determined by the electronic structure calculations of ITO-MoS_2_ with a *n*-type Schottky barrier height (*Φ*_n_) of ~ 0.2 eV^10^. However, the research on mixed phases based on TMDC heterostructures still requires an extended understanding of their properties and potential improvements in their fabrication.

Most of the research on mixed MoS_2−x_Se_x_ phases starts with single- and bi-layer systems obtained by mechanical exfoliation and manual layer stacking^11–13^ which is a methodology suitable for fundamental research but not scalable for large-area and low-cost production and integration with devices. Most metastable phases reported in the literature described Janus-like heterojunctions based on TMDC—a Janus heterojunction required substituting half the original chalcogen composition with a different one, commonly substituting S by Se or vice versa, and this substitution needs to occur in only one atomic layer. Their fabrication is also limited to the critically controlled environments required for their fabrication, and from these reports, its long-term stability and scalability can be under question. In contrast, proof of large-scale fabrication of MoS_2_ thin films has been reported by Muratore et al.^14^, Ramos et al.^2^, and others^15–17^, by using RF-sputtering, which is a low-cost method and already employed in commercial manufacturing and attain laminar colonies of 2H MoS_2_ grains oriented vertically in the <001> basal plane or <101> plane direction.

Remarkably, Conca et al. achieved doping a nanocrystalline silicon thin film with boron via an RF-sputtering co-deposit process with and ease of control over the boron concentration and the resulting thin film crystallinity^18^. Furthermore, co-deposit by RF sputtering dates back decades, when Stupp improved the tribological properties of MoS_2_ coatings as a function of co-deposited metals by RF-sputtering^19^. This work has been followed recently by other authors to enhance the catalytic properties of silver-decorated MoS_2_ nanoflakes^20^ and the performance of biosensors^21^. In addition, theoretical calculations on Janus heterostructures and related metastable phases are needed to provide new insights into mixed phases and their performance, like electron–phonon relations^22^, heterostructure coupling^9,23^, photocatalyst capacity^24^, and especially, thermodynamic stability. To date, no theoretical study compares Janus and random MoS_2−x_Se_x_ mixed phase in terms of thermodynamic stability and basic electronic properties at different concentrations (up to 33.33 at. % of selenium) to attain a MoSSe phase.

This work contributes to the research on mixed phases based on TMDC by demonstrating the ease of fabrication of a MoSSe thin film mixed phase and correlating these experimental data with density functional theory-based calculations. We start presenting the characterization of a MoSSe thin film co-deposited by RF-sputtering. Raman spectroscopy, time-of-flight secondary ion mass spectrometry, and scanning electron microscopy indicate a random distribution of sulfur and selenium atoms within the thin film. Next, we present the computation of the thermodynamic stability of a MoS_2−x_Se_x_ mixed phase in a Janus-like and random distribution from 0 at.% up to 33.33 at.% selenium concentration. Our results indicate that lattice expansion is related to the observed Raman shift, and the formation process of random MoS_2−x_Se_x_ meta-phase is more suitable for practical applications as semiconductors, where long-term stability is the main concern. We believe this information is essential to boost improvements in the fabrication methods of large-area and scalable mixed thin films using RF-sputtering.

## Results and discussion

### Fabrication of the MoS_2−__x_Se_x_ phase

**Figure 1:**
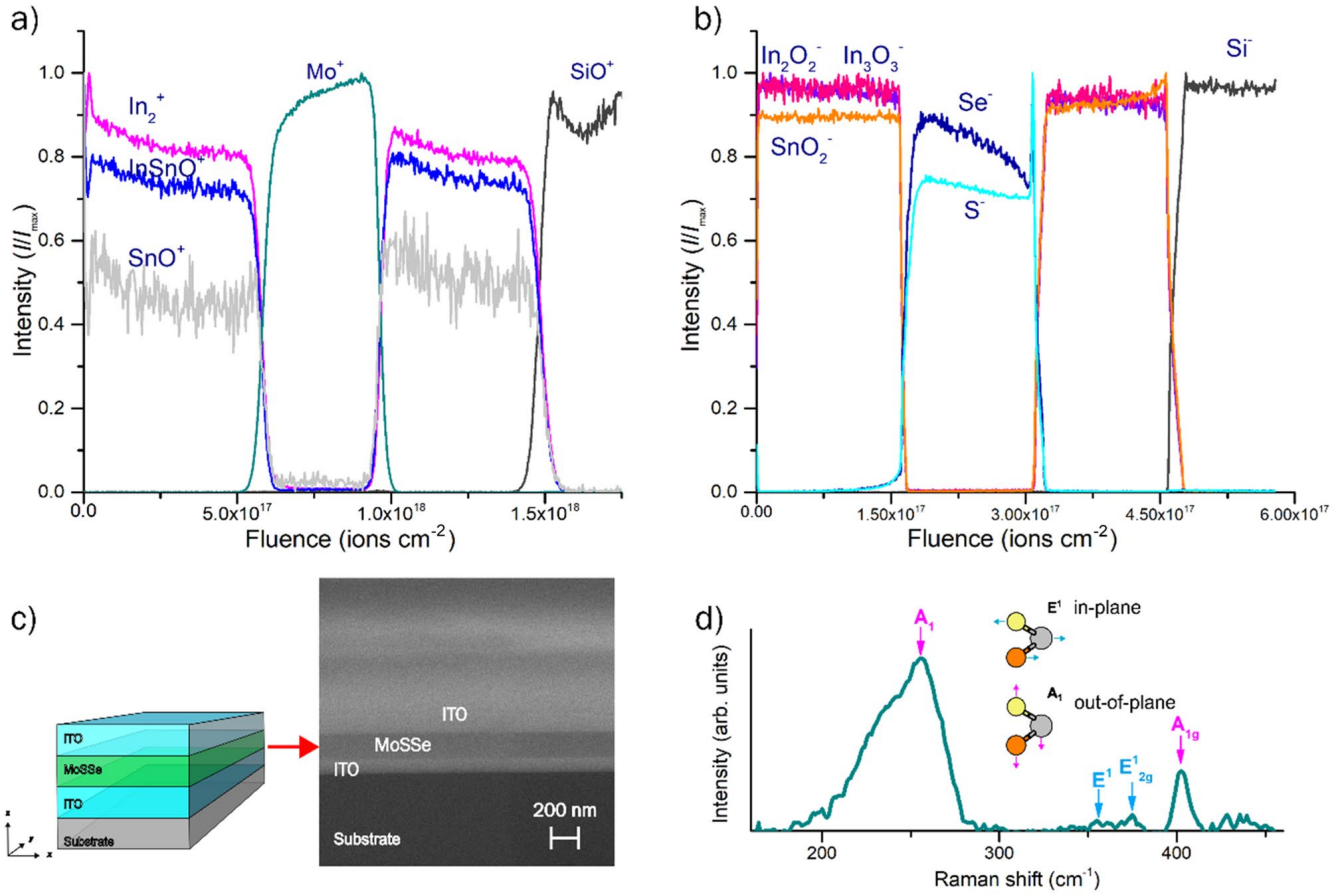
(**a**, **b**) Time-of-flight secondary ion mass spectrometry depth profile measurements for positive and negative ions, respectively. (**c**) Schematic description of the deposited multilayer arrangement and cross-section scanning electron microscopy image. (**d**) Raman spectra of the obtained MoSSe thin film showing the blue-shifted A^1^ and E^1^ corresponding to the MoSSe phase. Inset displays a schematic of the A^1^ and E^1^ Raman modes of MoSSe with gray balls denoting molybdenum atoms, and orange and yellow selenium and sulfur atoms, respectively.

From the co-deposition process of MoS_2−x_Se_x_ phase by RF-sputtering, we obtained a thin film of approximately 200 nm sandwiched between ITO layers (being approx. 150 nm thick). ToF–SIMS measurements confirm that our multilayer array consists of four distinctly different regions with sharp interface formation (Fig. 1). The presence of sharp interfaces between the top and bottom ITO layers with the MoS_2-x_Se_x_ phase in between is specified by the signals for In^2+^, InSnO^+^, and SnO^+^ ions are distinguished and decay exponentially as the Mo^+^ signals start to appear as depicted in Fig. 1a implying no intermixing between the ITO and the MoS_2−x_Se_x_ phase. In terms of selenium and sulfur, these measurements detected a homogeneous distribution within the fabricated sample, denoted by the signals from S^−^ and Se^−^ ions (Fig. 1b). Cross-section scanning electron microscopy (SEM) images present visual aspects of the ITO/MoS_2−x_Se_x_/ITO array (Fig. 1c), confirming a well-defined and sharp interface formation as previously reported^10,25^ and of comparable quality to those obtained by CVD methods^26^.

The Raman spectroscopy reveals that the film is homogenous and presents a degree of crystallinity (Fig. 1d). The sample presents vibrational modes at 402 cm^−1^ and 374.8 cm^−1^, which correspond to the A_1g_ and E^1^_2g_ vibrational modes of MoS_2_ and suggest the presence of pure Mo-S vibration in some regions (Supplemental Information Fig. S1). However, signals at 355 cm^−1^ and 255 cm^−1^ correspond to the blue-shifted E^1^ and A_1_ modes, respectively, due to the formation of the MoSSe phase^27,28^. The blue shift in the A_1_ vibrational mode is attributed to the out-of-plane change in symmetry caused by the integration of Se into the MoS_2_ matrix during the co-deposit, while the shift in E^1^ modes is related to the expansion of the lattice^29^ and will be discussed in the next section. Deconvolution of the Raman spectra shows a thin film with traces of MoSe_2_ signals, detectable by the A_1g_ Raman mode before 250 cm^−1^ alongside the shifted A_1_ modes of the MoSSe phase (Supplemental Information Fig. S2). Grazing incidence x-ray diffraction (GIXD) spectroscopy matches with our Raman analysis on the degree of crystallinity of the sample, indicating the presence of peaks at 13°, 33°, 54° and 56° which corresponds to the Bragg’s angle (2θ) of the planes (002), (101), (008) and (100) of 2H MoS_2_ and 2H MoSe_2_^30–32^ with P6_3_/mmc-194 space group, as shown in Fig. S3 from the Supplemental Information. The average crystal size was calculated to be ~ 75 nm (Table S1) in agreement with previous reports^2^ by using the Scherrer equation. Future studies can exploit low-energy methods like laser annealing^33^, annealing in a controlled atmosphere^15,26,34^, or ink printing^35^, integrated into the one-step process to fabricate mixed MoS_2−x_Se_x_ thin films with improved crystal quality for applications in energy harvesting and optoelectronics devices.

Compared to previous reports on RF-sputtering of MoS_2_ and MoSe_2_, we report the fabrication of a co-sputtered MoSSe phase under a low-temperature and high-power conditions for an smooth and homogenous deposit knowing that high RF power tends to facilitate the crystallization of MoS_2_^32^. The listed works in Table 1 also show a similar trend, indicating that for an optimal deposition of MoS_2_ by RF-sputtering, and probably most TMDC, enough kinetic energy is require to achieve the Mo^+4^ state and with this, an homogeneous crystallization^36^. This former argument is supported by our computational analysis presented in the next section. In our work, we increased the RF power for high deposition rates, but we also increased the working distance (see “Methods” section) to avoid the impact of high-energy sputtered material on the substrate. Furthermore, the literature also points out that deposition time has a crucial role in determining the crystallinity of the MoS_2_ thin film, as short deposition times (< 120 s) normally yield amorphous MoS_x_ phases or minuscule crystalline structures^30^. Then, the current course in physical vapor deposition (PVD) of TMDC, either by RF or direct current (DC), is to ease the fabrication of thin films avoiding subsequent high-energy annealing and post-sputtered treatments, aiming for one-step direct processes.

**Table 1:** Comparison of different MoS_2_ and MoSe_2_ fabrication methods and the resulting characteristics of the material.

Thin film material	Fabrication method	Fabrication parameters	Characteristics	References
MoSSe	RF sputtering (using 99.99% purity MoS_2_ and MoSe_2_ commercial targets), on thermally oxidized Si/SiO_2_ substrates	Power = 275 W Dwell time = 15 min Working pressure = 4 × 10^–6^ bar Substrate temperature = RT Working distance = 25 cm	Thin film Thickness = ~ 200 nm Homogenous sulfur and selenium distribution Sharp interface formation Blue-shifted E^1^ and A_1_ Raman modes at 355 cm^−1^ and 255 cm^−1^	*This work*
MoS_2_	RF sputtering on Si/SiO_2_ substrates	Power = 275 W Dwell time = 5 min Working pressure = 4 × 10^–6^ bar Substrate temperature = RT Working distance = N/A	Thin film Thickness = ~ 105 nm Highly crystalline MoS_2_ E^1^_2g_ and A_1g_ normal Raman modes at 378 cm^−1^ and 407 cm^−1^	^2^
MoS_2_	DC sputtering on glass and Si substrate	Power = 30 W, 40 W, 60 W Dwell time = 1 s–5 min Working pressure = 6.7 × 10^–6^ bar, 13.3 × 10 ^-6^ bar, 19.5 × 10^–6^ bar Substrate temperature = RT-400 °C Working distance = 5 cm	Thin film Thickness = 1–440 nm Nanocrystalline edge-rich E^1^_2g_ and A_1g_ normal Raman modes at ~ 376 cm^−1^ and ~ 408 cm^−1^ No direct relation between substrate and resulting crystallinity Higher crystallinity with higher deposition time	^30^
MoS_2_	RF sputtering (MoS_2_ target of 99.95% purity) on amorphous SiO_2_ and (002) oriented graphite substrates	Power = N/A Dwell time = N/A Working pressure = 2 × 10^–5^ bar Substrate temperature = 350 °C Working distance = 7 cm	Continuous MoS_2_ films Thickness = 3–6 molecular layers MoS_2_ growth parallel to the basal plane	^37^
MoS_2_	RF sputtering on SiO_2_/Si, quartz, and sapphire substrates	Power = 25 W Dwell time = 1, 3, 5 and 15 min Working pressure = 1.33 × 10^–5^ bar Substrate temperature = RT to 500 °C Working distance = N/A	Bilayer to few layer MoS_2_ domains Post-deposition annealing at 700 °C in a sulfur-rich atmosphere to improve crystallinity Improved carrier mobility	^16^
MoSe_2_	DC sputtering on quartz and Si substrates	Power = 75 W Dwell time = 4 min Working pressure = 6.7 × 10^–6^ bar Substrate temperature = RT Working distance = 5 cm	Thin film with wall-like structures Thickness = 325 nm Preferential growth along the *c*-axis E^1^_2g_ and A_1g_ normal Raman modes at ~ 242 cm^−1^ and ~ 284 cm^−1^	^31^
MoSe_2_	RF co-sputtering of Mo and Se targets with 99.99% purity on Si substrate	Power = 15–45 W for Mo/15 to 25 W for Se target Dwell time = N/A Working pressure = 1.33 × 10^–5^ bar Substrate temperature = N/A Working distance = N/A	Annealing improve material’s crystallinity Thickness = 200 nm Formation of MoO_3_	^38^
MoSe_2_	CVD using Selenium pellets (99.9%) and MoO3) (99%) powder as precursors on Si/SiO_2_ substrate and growth at 750 °C	Power = N/A Dwell time = 20 min Working pressure = N/A Substrate temperature = N/A Working distance = N/A	Triangle domains of MoSe_2_ Thickness = 0.8 nm E^1^_2g_ and A_1g_ normal Raman modes at ~ 239 cm^−1^ and ~ 301 cm^−1^	^39^

This benchmark is focused on large-scale and large-area sputtering reports of MoS_2_ and MoSe_2_ thin film fabrication and not limited to RF-sputtering only.

### Structural optimization of the MoS_2−x_Se_x_ phase

To obtain insights into the structural stability and possible formation routes, we considered a MoS_2−x_Se_x_ phase model using density functional theory (DFT) calculations. Details are provided in the “Methods” section. MoS_2_ crystallizes in the hexagonal P6_3_/mmc space group, and its structure consists of two MoS_2_ sheets oriented parallel to the (001) plane. From these calculations, it is found that as the concentration of selenium increases, the initial MoS_2_ lattice needs to expand in the random and Janus-like situations to accommodate the foreign ions and reach the structural optimization convergence. In the MoS_2−x_Se_x_ random phase at 33.33 at. % of selenium, a relative volume expansion (*ΔV*) of almost 7% is noted, while in the Janus-like situation the *ΔV* results 5.8% (see Table 2). The random phase at 33.33 at. % of selenium approaches more closely to the lattice parameter of 0.329 nm for 2H MoSe_2_^40,41^. The volume variation indicates a distortion from the pure MoS_2_ to the MoS_2−x_Se_x_ phase in the random situation and contrasts with the volume variation in the Janus-like, which is less abrupt. The volume variation in the Janus-like model is in line with previous reports, where a low lattice distortion occurs in the experimental Janus MoSSe monolayer^42^.

**Table 2:** Formation energy (*E*_form_/eV atom^−1^), substitutional energy (*E*_subst_/eV atom^−^), substitutional energy with vacancy defects (*E*_subst-V_/eV atom^−1^), resulting lattice constant (***a***), lattice parameters (*a*, b, and c), volume (*V*), and relative volume expansion (*ΔV*) of the random and Janus-type MoS_2−x_Se_x_ phases at different concentrations of Se. Negative values of *E*_form_, *E*_seg_, and *E*_seg-V_ designate a thermodynamically favorable process.

Se at. %	*E*_form_/eV atom^−1^	*E*_subst_/eV atom^−1^	*E*_susbt-V_/eV atom^−1^	***a***/nm	*a*/nm	*b*/nm	*c*/nm	*V*/nm^*3*^	*ΔV*
Random MoS_2−x_Se_x_									
0.0	–	–	–	0.3167	1.2668	1.2668	1.2668	1.7345	0.0%
4	− 7.515	0.036	0.332	0.3174	1.270	1.270	1.254	1.750	0.92%
8	− 7.479	0.072	0.663	0.3182	1.273	1.273	1.259	1.766	1.81%
16	− 7.407	0.144	1.326	0.3196	1.279	1.279	1.270	1.797	3.49%
33.3	− 7.263	0.288	2.651	0.3227	1.291	1.291	1.285	1.855	6.49%
Janus MoS_2−x_Se_x_									
4	8.572	16.123	16.419	3.1875	1.275	1.275	1.248	1.756	0.47%
8	8.608	16.159	16.750	3.1948	1.278	1.278	1.253	1.770	1.78%
16	− 7.406	0.145	1.327	3.1968	1.279	1.279	1.266	1.792	3.33%
33.3	− 7.259	0.292	2.656	0.3229	1.292	1.292	1.275	1.842	5.84%

We attribute the increase in the volume of the supercell as a direct consequence of the change in the bonding distance between molybdenum and the chalcogen ions. In the random phase, the Mo-S bond distance (*d*_Mo-S_), presents a contraction from 0.2406 nm to 0.2405 nm in most cases, a minor relative contraction of 0.04%. The Mo-Se bond distance (*d*_Mo-Se_) shows contractions up to 0.08%, recalling the *d*_Mo-Se_ in pure MoSe_2_ resulted in 0.25322 nm. In the Janus distribution, an expansion of 0.17% of *d*_Mo-S_ resulted after the structural optimization, while the *d*_Mo-Se_ presents a contraction close to 0.1% concerning the pure 2H phase in both cases. However, as the *d*_Mo-Se_ is significantly larger than *d*_Mo-S_, about 4%, we found a significant change in the resulting volume of the MoS_2−x_Se_x_. This is attributed to the rearrangement of charge from disturbed Mo *d* orbitals and *p* orbitals as reported previously^6,43^ and possible electron density change in the 4*d* orbital due to lower electronegativity of Se over S. This uneven charge distribution may lead to local piezoelectricity as reported in other studies on Janus heterojunctions^7,44,45^.

This change in the bonding distance agrees to previous reports^27^ on Janus MoSSe systems and may be related to the resulting blue shift in the E^1^ and A_1_ Raman modes, which relates with our experimental observations presented earlier. The A_1_ Raman modes represent the out-of-plane vibrational modes, perpendicular to the (001) basal plane, but selenium and sulfur have different bonding distances with molybdenum, affecting the symmetry along such planes and thus, the resulting vibrational mode. Similarly, the in-plane E^1^ vibrational modes (alongside the atomic plane of the S-Mo-S distribution) will have differences compared to the pure 2H phase due to the included selenium. Even though in our case we focused on bulk structures, we expect a comparable explanation for monolayers and two-dimensional (2D) systems.

### Thermodynamic stability of the MoS_2−x_Se_x_ phase

**Figure 2:**
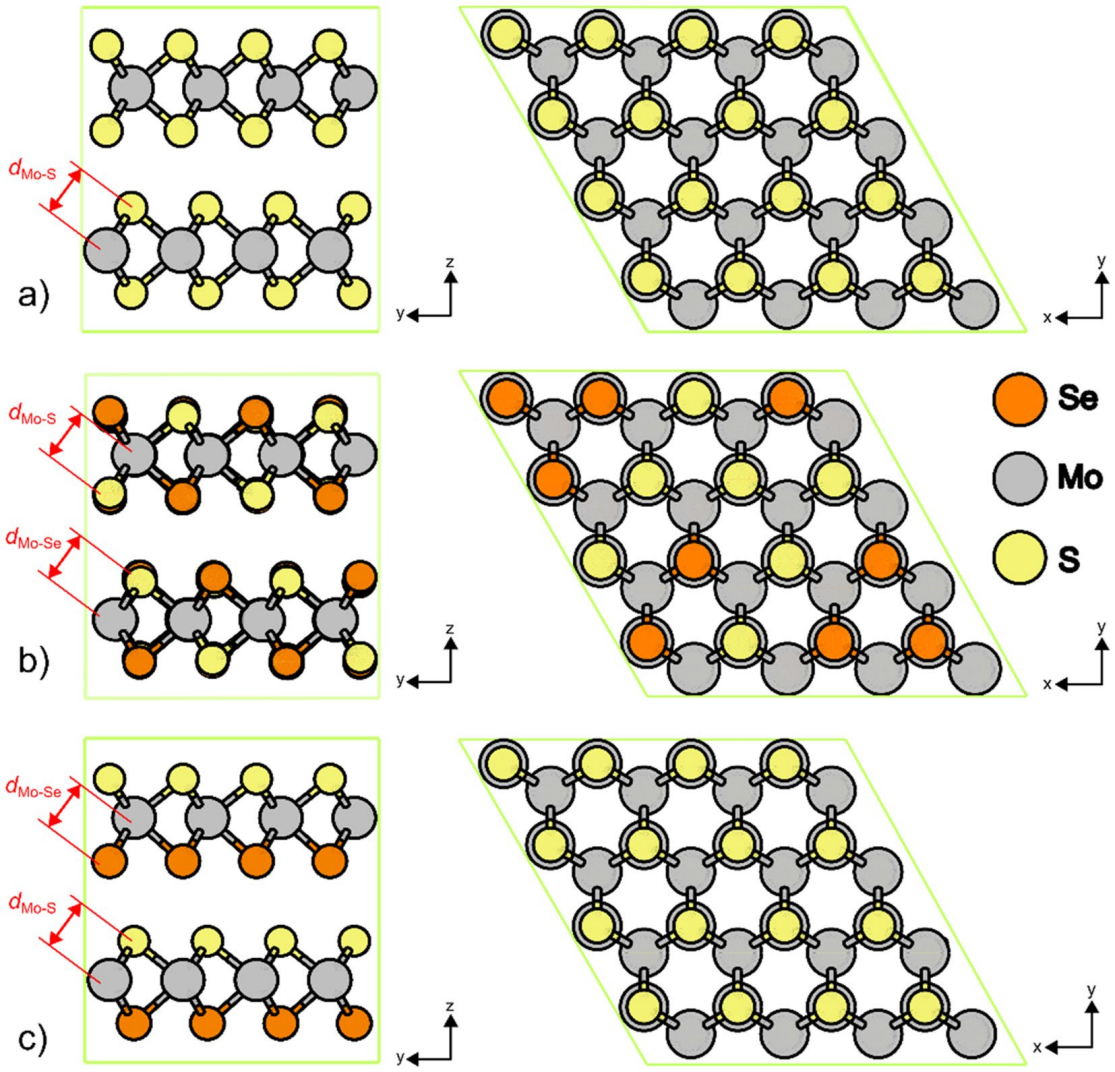
(**a**) Schematic of the 2H-MoS_2_ supercell, (**b**) random MoS_2−x_Se_x_ supercell at *x* = 1, and (**c**) Janus-type MoS_2−x_Se_x_ supercell at *x* = 1. All MoS_2-x_Se_x_ models were created using a 2H-MoS_2_ 4 × 4 × 1 supercell containing 32 molybdenum atoms and 64 sulfur atoms. *Color code:* yellow is sulfur, orange is selenium and grey corresponds to molybdenum atoms.

The formation energy (*E*_form_) -for MoS_2−x_Se_x_ phase for various selenium concentrations- indicates that at low and high selenium concentrations, the lattice can favorably accommodate these ions in the random distribution. Figure 2 describes the atomistic model used for the DFT computations. For the Janus-type phase, at low selenium concentrations, the system reaches a low entropy state, and the process becomes endothermic. Under these circumstances, the lattice shows a lattice expansion situation quantified by a *ΔV* of 1.78%. However, after surpassing a selenium concentration of 8 at.% the Janus-like lattice reaches favorable conditions of formation, expressed by the exothermic values of *E*_form_. The clear presence of a threshold value for the formation of the Janus-like phase indicates that it would not be a feasible process unless enough energy is provided to overcome this energy barrier. On the other hand, the random phase shows an exothermic tendency, regardless of the selenium concentration. This analysis indicates that a co-deposit process would favor the formation of the random MoS_2−x_Se_x_ phase.

**Figure 3:**
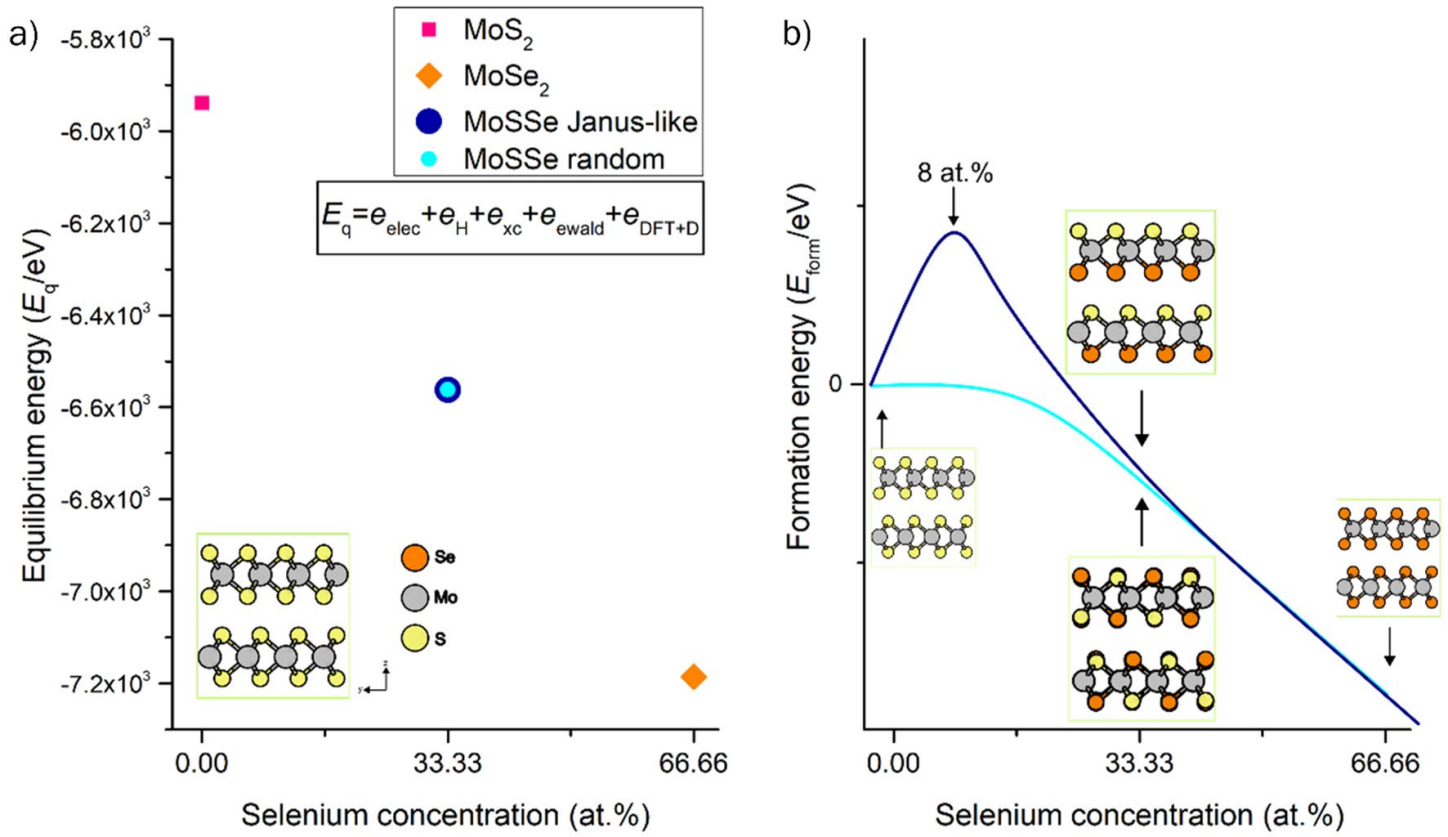
(**a**) Computed equilibrium energy (*E*_q_) for pure 2H MoS_2_, 2H MoSe_2_, Janus, and random MoS_2−x_Se_x_ at a Se concentration of 33.33 at.%. The random MoS_2-x_Se_x_ phase at x = 1 reaches almost the same entropy state as the Janus-like phase. *E*_q_ computed by Quantum Espresso code includes a one-electron energy part (*e*_elec_), the Hartree contribution (*e*_H_), the exchange–correlation energy (*e*_xc_), the Ewald contribution (*e*_ewald_), and the dispersion correction part by DFT + 3 (*e*_DFT+D_). (**b**) Estimated energy profile for the transition from pure MoS_2_ to pure MoSe_2_ having a random and Janus-like phase. The fabrication of MoSSe Janus-like would be limited by an energy barrier at 8 at. % of selenium. Arrows point to the location of their relative energy of formation.

In terms of the site specific substitution of S by Se—denoted by the substitutional energy, *E*_subst_—we observe that for the random phase, the process is endothermic in all ranges of selenium concentrations up to 33.3 at.% having energy demands below 0.3 eV, which is comparable to the proposed activation energy for MoS_2_ crystallization of 0.7 eV^46^. In this situation, as the concentration of selenium increases, the energy demand also increases as the systems need to accommodate foreign Se atoms into the initial matrix. For the Janus-like phase, the process is not favorable until a threshold value of 8 at.% selenium concentration is reached. In the latter situation, the presence of an energy barrier becomes noticeable; for concentrations below 8 at. % of selenium, the system has no tendency to exchange sulfur with selenium with *E*_subst_ values above 16 eV. After surpassing this threshold value, the process becomes favorable and behaves similarly to the random phase, with substitution energies below 0.3 eV. This suggests that subsequent substitution is self-maintained in the Janus-type phase. Previous reports have hinted towards the critical stability of Janus MoSSe monolayers between 700 °C and 800 °C^47,48^. Lu et al.^27^ report a randomized MoSSe phase achieved above 600 °C, which supports our statement that MoSSe Janus-type structures are indeed low entropy systems in a metastable state. The Janus-like and random phases reach almost an equal value of equilibrium energy (Fig. 3a), meaning both phases are located at the same low entropy point, and with this, the same metastable state located between pure 2H MoS_2_ and pure 2H MoSe_2_ as depicted in Fig. 3b. Nevertheless, the low entropy state of the Janus-type is determined by the highly ordered distribution of selenium and sulfur ions, related to the observed increase in *E*_subst_ as mentioned before.

Sulfur vacancies might be present in sputtered MoS_2_^49^, consequently on MoSe_2_ as well, due to the higher vapor pressure of the chalcogen species^46^. To explore the role of S vacancies as lattice defects in the substitutional process of Se into MoS_2_, we computed the values of *E*_subst_ but added an energy contribution for sulfur vacancy formation. The computed formation energy of a sulfur vacancy (*E*_V_) is 7.09 eV, meaning it is an endothermic process and is within the range of defect formation in MoS_2_^37,50,51^. The latter was added to the previous *E*_subst_ to get an energy of substitution with an S vacancy formation (*E*_subst-V_) and expressed as $E_{subst - V} = E_{seg} + n_{V} E_{V}$, where *n*_*V*_ is the number of S vacancies needed to accommodate the targeted Se concentration. The difference in our computed energy of vacancy formation with previous studies reporting the effect of S vacancies in MoS_2_ is attributed to the addition of long-range dispersion correction in our study and the fact that we are using a crystal model rather than monolayer models. Besides, using crystal models helped to reach lower entropy phases compared to monolayers, i.e., lower surface energy. From this analysis, a clear difference between the Janus and the random MoS_2−x_Se_x_ phase becomes noticeable. For the Janus-like phase, the inclusion of Se atoms in a MoS_2_ lattice with *n*_V_ sulfur vacancies is not favorable to occur below a Se concentration of 8.3 at. %, after this tipping point the process follows a self-maintained behavior (Table 2) and the energy demand for creating S vacancies is compensated. For the random MoS_2-x_Se_x_ phase, the contrary occurs, as the energy demand remains in the endothermic regime but is doable in terms of energy supply. This indicates that the fabrication of Janus-like phases is locked or inhibited by this energy barrier and explains the experiments performed by Lu et al.^27^ and Li et al.^52^ and the critical part of the sulfur vacancy creation and post-selenization process (Fig. 3).

### Electronic structure of the MoS_2−x_Se_x_ phase

**Figure 4:**
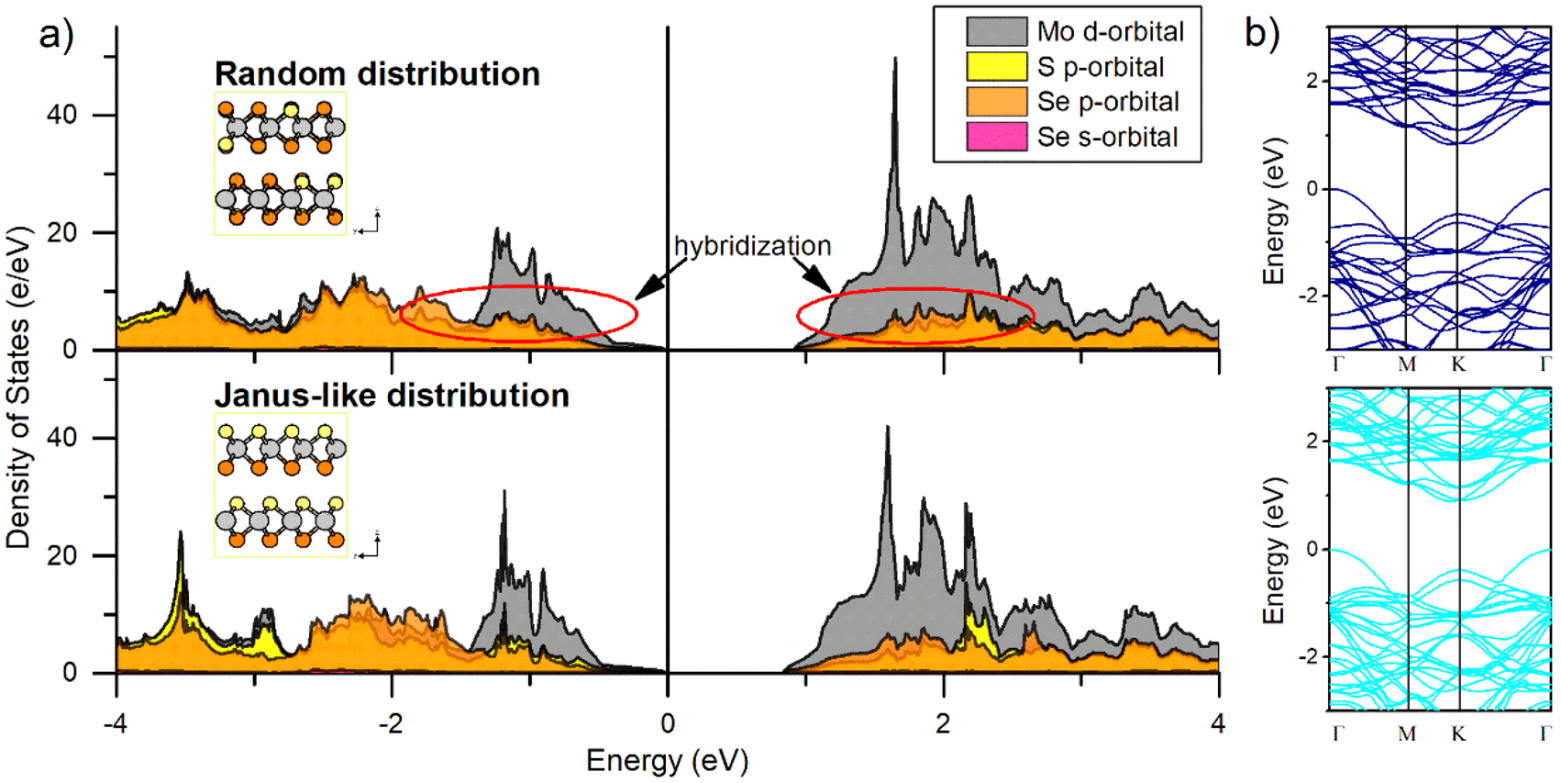
(**a**) Computed projected density of states (PDOS) for the MoSSe in a random and Janus-like distribution. The Fermi level is located at 0 eV. The MoSSe phase having a random distribution presents a higher degree of orbital hybridization between the S p- and Se p-orbital, indicated by the similarities in the curvatures of the pDOS around the Fermi level. (**b**) Computed band structure for the random and Janus-like distribution in the Γ-Μ-Κ-Γ path. The estimated band gap of both situations is close to 0.9 eV.

To get insights into the type of bonding between the chalcogen atoms and the molybdenum, we computed the electronic structure for the random and the Janus-like distribution. First, the projected density of states (PDOS) indicates the strong metallic influence around the Fermi level, characterized by the high contribution of the molybdenum *d* orbitals in all situations like in the case of MoS_2_ and MoSe_2_^10,41,53^. Second, the contribution of the sulfur and selenium *p* orbitals in the random distribution has a pronounced overlap around the Fermi level, indicated by the close similarities in the sulfur and selenium DOS curves’ distribution. This overlap is less pronounced in the Janus-like distribution compared to the latter, especially at – 1 eV and after 2 eV (Fig. 4). The PDOS analysis suggests a higher degree of coupling between the sulfur and the selenium ions when the MoSSe phase is reached in the random distribution, correlating the resulting contraction in the *d*_Mo-S_ and *d*_Mo-Se_ as described in previous sections. Inside the Janus-like distribution, a strong peak at − 1.2 eV suggests a hybridization of sulfur and selenium *p* orbitals and molybdenum *d* orbitals, primordially attributed to the in-plane distribution of the chalcogen atoms and alignment of the *p*_*x*_, *p*_*y*,_ and *p*_*z*_ symmetries. Lastly, the computed band structure reveals that the MoSSe phase remains with its semiconducting properties and a reduced indirect band gap close to 0.9 eV in both situations. Such reduction in the band gap agrees with what was observed by Li and coworkers, where a graded MoS_2(1−x)_Se_2x_ nanosheet showed a variable band gap ranging from 1.8 eV to 1.6 eV as the selenium concentration increased^48^. The latter potentially implies that the MoS_2−x_Se_x_ mixed phase could work as a research platform for bandgap engineering of TMDC thin films.

## Conclusions

The co-deposited thin film exhibits blue-shifted A_1_ and E^1^ Raman modes at 355 cm^−1^ and 255 cm^−1^, which correspond to the Raman modes of the MoSSe phase and are evidence of lattice expansion. Time-of-flight secondary mass spectrometry shows an even distribution of selenium and sulfur ions throughout the thin film and evidenced the fabrication of sharp interfaces with the encapsulated layers. Our density functional theory calculations demonstrate that the formation of the random MoS_2−x_Se_x_ phase is thermodynamically favorable compared to the Janus-like phase, the latter hindered by an energy barrier below 8 at.% selenium concentrations. Interestingly, both mixed phases reached similar metastable states, denoted by their corresponding equilibrium energy. However, they presented different lattice expansion, above 7% for the random distribution and 5% for the Janus-like, attributed to different orbital’s reallocation in Mo-S and Mo-Se bonds. These results display outstanding ease of fabrication of MoS_2-x_Se_x_ mixed phase with a random distribution of chalcogens by RF-sputtering. We believe that this report provides critical insights that can enhance the fabrication methods of large-area and scalable MoS_2−x_Se_x_ mixed phase in the future.

## Methods

### RF-sputtering of MoS_2−__x_Se_x_ phase

The co-deposition by RF magnetron sputtering of the MoS_2−x_Se_x_ phase was completed using a Kurt J. Lesker PVD-75 instrument, which is equipped with three holders and these can open simultaneously during the deposition process without breaking the vacuum. ITO, MoS_2_ and MoSe_2_ targets were mounted separately inside each holder. The substrate was a thermally oxidized Si/SiO_2_ with 25.4 mm in diameter mounted within a working distance of 20 cm and no heating was applied to the substrate during the co-deposition process. The system was ready for the deposition once a vacuum of 1 × 10^–9^ bar was reached. After this, a flow or Ar^+^ ions was introduced into the main chamber setting a working pressure of 4 × 10^–6^ bar. A layer of indium-tin-oxide (ITO) was first deposited to enhance the adhesion of the MoS_2-x_Se_x_ phase to the substrate using a commercial target of In_2_Sn_2_O_7_ (99.9% purity and 76.2 mm in diameter) for 1800 s at 145 W of RF power at a frequency of 13.56 MHz to achieve the ~ 150 nm layer thickness. Subsequently, the MoS_2−x_Se_x_ phase was deposited by simultaneous sputtering of MoS_2_ and MoSe_2_ targets (99.99% purity and 76.2 mm in diameter) for 1000 s at 275 W of RF power at a frequency of 13.56 MHz, aiming for a MoSSe phase with a layer thickness of approximately 200 nm. Finally, a layer of ITO was deposited on top of the MoSSe phase to protect the material from environmental degradation without breaking the vacuum with the same parameters described before.

### Cross-section, time-of-flight secondary ion mass spectrometry, Grazing incidence x-ray diffraction, and Raman spectroscopy

Cross-section images were acquired using a scanning electron microscope ZEISS Auriga 60 high-resolution dual beam equipped with an ion gun to qualify the interface formation of our co-deposited thin film. Images were recorded using the InLens detector at 20 kV. A ToFSIMS5-100 (ION-TOF GmbH) instrument was used for the time-of-flight secondary ion mass spectrometry (ToF-SIMS) with the aim of resolving the elemental composition of the thin film. This spectrometer is equipped with a Bi cluster primary ion source (field emission from liquid Bi wetting a tungsten tip) and a reflectron type time-of-flight analyzer. Ultra-high vacuum (UHV) base pressure during analysis was < 3 × 10^–8^ mbar. For high mass resolution the Bi source was operated in the “high current bunched” mode providing short Bi^+^ primary ion pulses at 25 keV energy, a lateral resolution of approx. 4 μm, and a target current of 1.4 pA. The short pulse length of 1 ns allowed for high mass resolution (8000 m/∆m). The primary ion beam was scanned across a 250 µm^2^ × 250 µm^2^ field of view on the sample, and 64 × 64 data points were recorded. For depth profiling a dual beam analysis in interlaced mode was performed. The sputter gun (operated with Cs^+^ or O_2_^+^ ions, 2 keV, scanned over a concentric field of 500 µm^2^ × 500 µm^2^, and target current of 180 nA and 600 nA, respectively) was applied to erode the sample. No further sample preparation was required for this measurement. Grazing incidence x-ray diffraction (GIXD) spectroscopy was collected using a Panalytical X-Pert system with a CuK_α_ radiation source at 40 kV and 35 mA at room temperature. The grazing incidence angle was set at 5° < θ < 60° and step size of 0.05° using a graphite flat crystal monochromator. Raman measurements were used to discern the crystallinity of the thin films and were run on a Renishaw inVia Raman microscope using a laser excitation wavelength (*λ*_e_) of 532 nm, a laser power of approx. 2 mW, and a 100x NA0.85 objective lens. All Raman measurements were taken at room temperature as well.

### Computational details

Our DFT calculations were carried out utilizing the Quantum Espresso^54,55^ package, which solves the Kohn–Sham equations by a plane-wave method. For the exchange-correlations term, the generalized gradient approximation (GGA) and the Perdew–Burke–Ernzerhof (PBE) options were chosen. The electrons’ distribution were described by the optimized Vanderbilt pseudopotentials^56^ provided through the SSSP package in its 1.2.1 version^57^. To account for the long-range forces inherently present in layered materials, we included the long-range dispersion correction DFT-D3 as described by Grimme et al.^58^ for an accurate description of the material. Structural visualization of the models was assisted by the VESTA^59^ and XCrysden^60^ codes. For the structural optimization calculations, the plane-wave cutoff energy was set to 470 eV, while the convergence criterion of ionic minimization was achieved when all forces were smaller than 5.1 × 10^–2^ eV nm^−1^ and the total energy changes less than 1.3 × 10^–2^ eV atom^−1^ in two consecutive self-consistent field steps. Additionally, a k-point set of 4 × 4 × 4 was used to sample in the Brillouin zone. During the structural optimization of the MoS_2−x_Se_x_ phases, all atoms were able to move freely, and the lattice dimensions were not fixed. For the electronic structure calculations, an increased plane-wave cutoff energy of 544 eV was used along with a denser k-point set of 10 × 10 × 10.

All models started with an optimized MoS_2_ unit cell with space group P6_3_/mmc and lattice constant ***a*** = ***b*** = 0.3168 nm, ***c*** = 1.249067 nm, ***α*** = ***β*** = 90°, and **γ** = 120°, with a Mo-S bond distance (*d*_Mo-S_) of 0.2406 nm. The ionic state of S and Se inside the lattice is as S^2−^ and Se^2−^, both in a three-coordinate geometry bonded to three equivalent Mo^4+^ ions, the latter remaining in trigonal prismatic coordination. We created a 4 × 4 × 1 supercell from this model containing 32 molybdenum atoms and 64 sulfur atoms. This ensured modeling a wide variety of selenium concentrations avoiding self-interactions and achieved a robust and reliable model. For this study, we considered the evolution of the MoS_2−x_Se_x_ phase to occur from a selenium concentration of 4 at.% up to a 33.3 at. %, corresponding to a 1:1 relation between S and Se.

Two types of MoS_2−x_Se_x_ phases were considered, the first in a random configuration and the second in a Janus-type configuration. For the random MoS_2−x_Se_x_ phase, the selenium atoms substituted sulfur atoms and were distributed randomly throughout the supercell model. For the Janus MoS_2-x_Se_x_ phase, the selenium atoms replaced sulfur atoms in only one atomic layer of the MoS_2_ structure as depicted in Fig. 1. The stability analysis of these two atomic arrangements first considered the formation energy per atom, (*E*_form_/eV atom^−1^), of the different MoS_2−x_Se_x_ phases, mimicking a co-deposit process, where all the atomic species converged to the formation of the targeted MoS_2-x_Se_x_ phase. This was computed using the formula:

$$E_{{{\text{form}}}} = \frac{1}{N}\left( {E_{{{\text{MoS}}_{{2 - {\text{x}}}} {\text{Se}}_{{\text{x}}} }} - \mathop \sum \limits_{i = 1}^{N} \mu_{i} } \right)$$

where $E_{{{\text{MoS}}_{{2 - {\text{x}}}} {\text{Se}}_{{\text{x}}} }}$ is the resulting enthalpy of the phase from the structural optimization calculations, *N* is the total number of atoms, and $\mu_{i}$ is the chemical potential of the $i$th atom in the mentioned phase; this latter parameter was considered as the bulk energy of the atom. This approach considers that the distribution of selenium and sulfur atoms have an equal probability and that no other type of defect is left at the supercell afterward. Secondly, we were interested in the case where a possible inclusion of Se might occur into the MoS_2_ lattice considering initially a pristine MoS_2_ supercell and ultimately derive in the formation of the targeted MoS_2-x_Se_x_ phase. For this scenario, the substitutional energy per atom (*E*_seg_/eV atom^−1^) was computed as follows:

$$E_{{{\text{subst}}}} = \frac{1}{N}\left( {E_{{{\text{MoS}}_{{2 - {\text{x}}}} {\text{Se}}_{{\text{x}}} }} - E_{{{\text{MoS}}_{2} }} + \mathop \sum \limits_{i = 1}^{n} \mu_{i}^{r} - \mathop \sum \limits_{i = 1}^{m} \mu_{i}^{s} } \right)$$

where *E*_MoS2_ is the energy of the 4 × 4 × 1 defect-free MoS_2_ supercell. Here, *µ*^r^ is the chemical potential of the *i*th-atom that was substituted or released from its original position, and *µ*^s^ is the chemical potential of the *i*th-atom that substitutes the released atoms. As in the previous case, we consider that the occupation of sulfur positions by selenium atoms has an equal probability and that no other type of defect exists at the supercell before or afterwards.

### Supplementary Information

Supplementary Information.

## Data Availability

The data that support the findings of this report are available from the corresponding authors upon reasonable request.
