# Supplementary material for: One-step sputtering of MoSSe metastable phase as thin film and predicted thermodynamic stability by computational methods
Source: Sci Rep. 2024 Mar 26;14:7104. doi: 10.1038/s41598-024-57243-3 (PMC10966109; doi:10.1038/s41598-024-57243-3)
Supplement: Supplementary file 1 — Supplementary Information. [file 41598_2024_57243_MOESM1_ESM.docx]

**Supplemental Information**

**One-step sputtering of MoSSe metastable phase as thin film and predicted thermodynamic stability by computational methods.**

Oscar A. López-Galán^1,2*^, Torben Boll^3^, John Nogan^4^, Delphine Chassaing^3^, Alexander Welle^3,5^, Martin Heilmaier^2^, and Manuel Ramos^6^

^1^Institute of Nanotechnology (INT), Karlsruhe Institute of Technology (KIT), Hermann-von-Helmholtz-Platz 1, 76344 Eggenstein-Leopoldshafen, Germany

^2^Institute for Applied Materials and Materials Science (IAM-WK), Karlsruhe Institute of Technology (KIT), Engelbert-Arnold-Str. 4, D-76131 Karlsruhe, Germany

^3^Karlsruhe Nano Micro Facility (KNMF), Karlsruhe Institute of Technology (KIT), Hermann-von-Helmholtz-Platz 1, 76344, Eggenstein-Leopoldshafen, Germany

^4^Center for Integrated Nanotechnologies, Sandia National Laboratories, 1101 Eubank Bldg. SE, Albuquerque, NM 87110, USA

^5^Institute of Functional Interfaces (IFG), Karlsruhe Institute of Technology (KIT), Hermann-von Helmholtz-Platz 1, 76344 Eggenstein-Leopoldshafen, Germany

^6^Departamento de Física y Matemáticas. Instituto de Ingeniería y Tecnología. Universidad Autónoma de Ciudad Juárez. Avenida del Charro #450 N, Ciudad Juárez 32310, Chihuahua, México

***Corresponding author:** [**oscar.galan@partner.kit.edu**](mailto:oscar.galan@partner.kit.edu)**;** [**manuel.ramos@uacj.mx**](mailto:manuel.ramos@uacj.mx)

***

***

Figure S1. Raman spectra of a multilayer MoS_2_/MoSe­_2_ arrangement showing the defined Raman modes for MoS_2_ and MoSe_2_. A Si/SiO_2_ substrate was used and the signal for Si appears in the spectra.


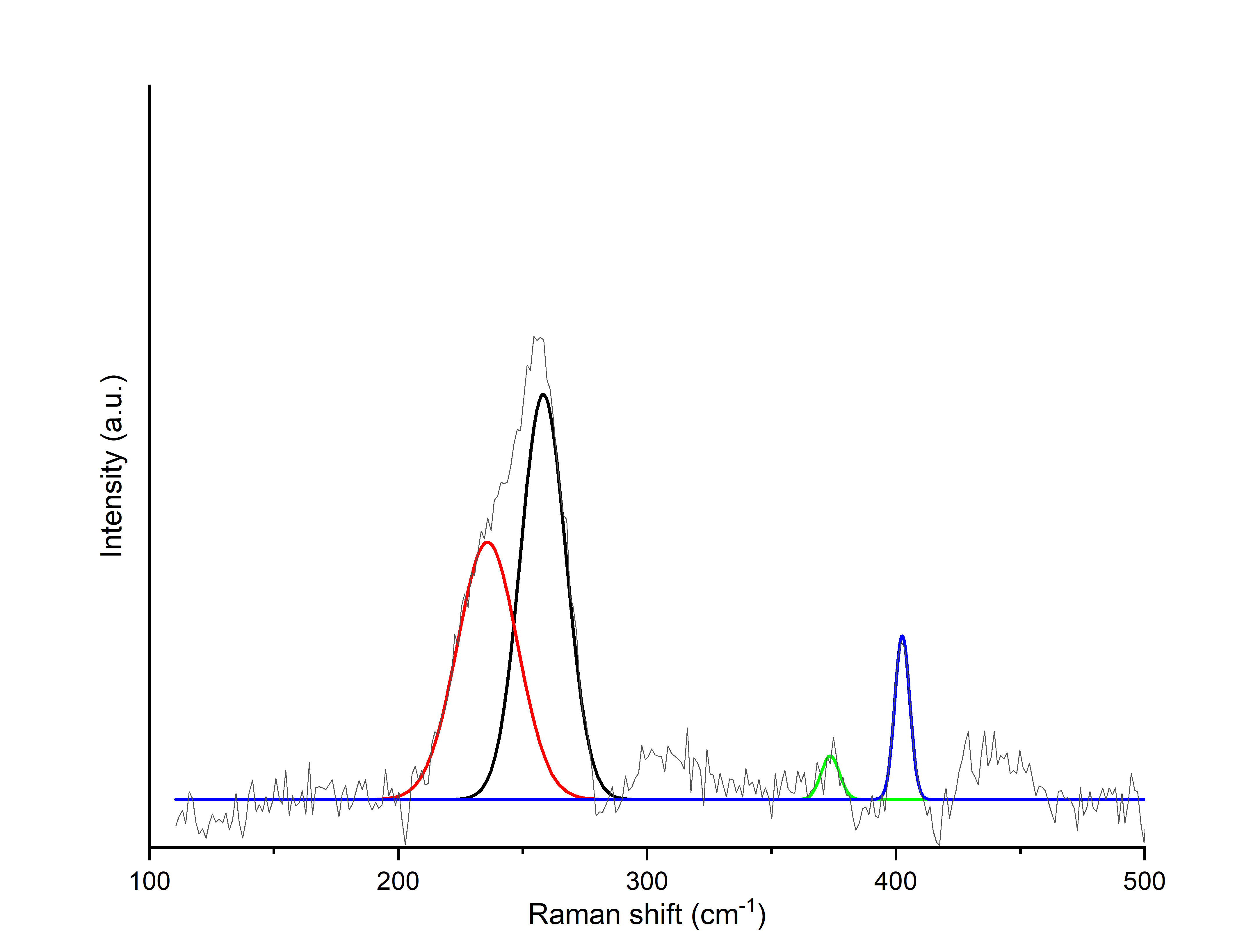


Figure S2. Deconvolution of the Raman spectra of the MoSSe phase showing the existence of a peak at c.a. 240 cm^-1^ and 260 cm^-1^ attributed to a blue-shift in the E^1^ and the presence of a A_1_ from MoSe_2_, respectively.


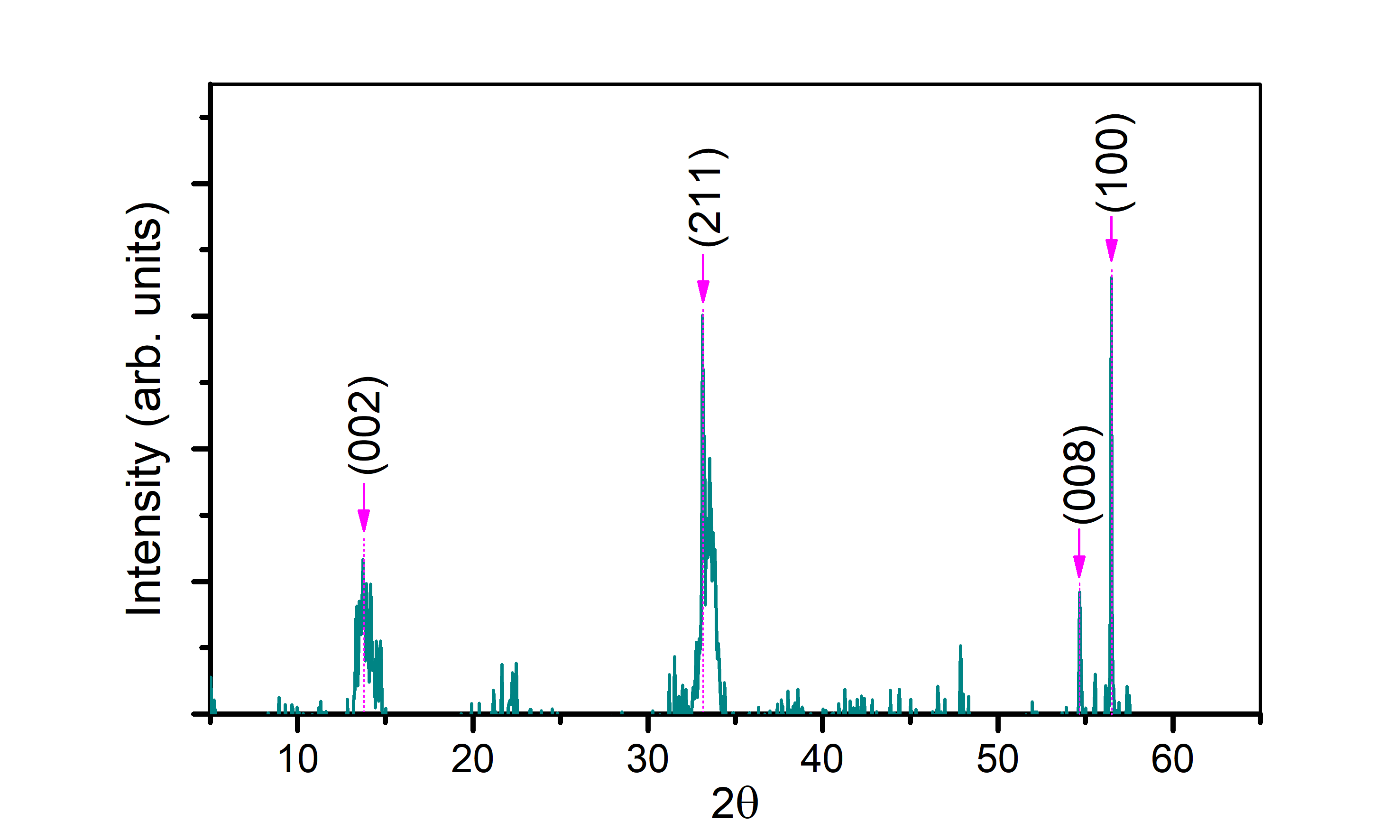


Figure S3. Grazing incidence x-ray diffraction spectroscopy of the sample with principal diffraction peaks at Bragg’s angle of 13°, 33°, and 56° corresponding to the (0 0 2), (2 1 1), and (1 0 0) planes of 2H MoS_2_.

Table S1. Grain size (*τ*) calculation using the Scherrer equation as $\tau=\frac{K\lambda}{\beta\cos\theta}$, being $K$ the shape factor equal to 0.9, $\lambda$ the wavelength of the x-ray radiation, $\beta$ the is the line broadening at half the maximum intensity and $\theta$ the angle.

| 2θ (°) | 2θ (rad) | FWHM | Grain size (*τ*/m) |
| --- | --- | --- | --- |
| 13.9886 | 0.244147128 | 8.78318 | 9.51× 10^-10^ |
| 33.15156 | 0.578603874 | 0.13475 | 6.42× 10^-8^ |
| 54.68869 | 0.954497704 | 0.09033 | 1.03× 10^-7^ |
| 56.47551 | 0.985683596 | 0.07169 | 1.31× 10^-7^ |
| Average grain size= | | | 7.5 × 10^-8^ |
